# Comparative study of the effects of VBT training with different velocity loss thresholds on lower limb explosive force of adolescent sprinters

**DOI:** 10.3389/fphys.2025.1746516

**Published:** 2026-01-27

**Authors:** Jingmiao Wang, Zhuo Zeng, Quanhong Lu, Song Yuan, Yunmei Chai

**Affiliations:** 1 School of Sport Training, Chengdu Sport University, Chengdu, China; 2 School of Strength and Conditioning Training, Beijing Sport University, Beijing, China

**Keywords:** movement velocity feedback, neuromuscular adaptation, resistance training, velocity-based training, youth athletes

## Abstract

**Objectives:**

This study aimed to investigate the effects of velocity-based training (VBT) on lower-limb explosive performance in adolescent sprinters and to compare the training adaptations induced by different velocity loss thresholds (VLT).

**Methods:**

Forty-five male adolescent sprinters were randomly assigned to three experimental groups that trained with VLT of 10% (G1), 20% (G2), and 30% (G3), respectively. All participants completed a 6-week VBT squat program performed twice per week at an intensity of 80% 1RM, with a fixed total volume of 20 repetitions per session. The session rating of perceived exertion (sRPE) was used to monitor subjective fatigue. Pre- and post-intervention tests included 30 m sprint performance, squat 1RM, countermovement jump (CMJ) height and relative peak power, and drop jump (DJ) reactive strength index (RSI). Data were analyzed using one-way ANOVA and paired-sample t-tests.

**Results:**

After 6 weeks of training, all groups showed significant improvements in squat 1RM, 30 m sprint performance, CMJ height, CMJ relative peak power, and DJ RSI (p < 0.05). Between-group comparisons revealed no significant differences in 1RM improvement (p > 0.05), whereas the 10% VLT group demonstrated significantly greater enhancements in CMJ height, CMJ relative peak power, 30 m sprint performance, and RSI compared with the 30% VLT group (p < 0.05). The overall trend in performance gains was consistent: 10% VLT > 20% VLT> 30% VLT. Monitoring data showed that sRPE values increased significantly with higher VLT (p < 0.001), indicating that lower VLT settings effectively reduced fatigue accumulation.

**Conclusion:**

VBT is an effective method for improving lower-limb explosive performance in adolescent sprinters. Under fixed training volume conditions, applying a lower VLT (e.g.,10%) produces superior training outcomes, likely due to reduced fatigue, maintenance of higher movement velocity and power output, and enhanced neuromuscular adaptations. Coaches are therefore advised to prioritize lower VLT when designing VBT programs aimed at developing explosive strength in youth sprinters.

## Graphical Abstract

**Figure:**
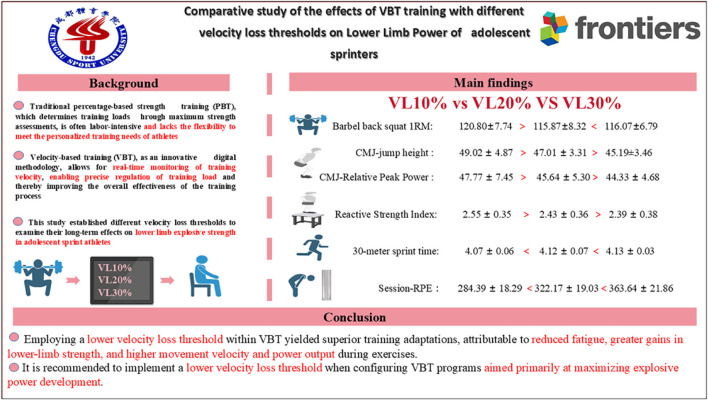

## 1 Introduction

The development of lower-limb explosive power is essential for elite sprinters (Eynon et al., 2013). Nevertheless, resistance training (RT) models such as percentage-based training (PBT) show limitations in terms of precision of load monitoring and individualization (Jiménez-Reyes et al., 2019). In response, velocity-based training (VBT) has emerged as a superior alternative. It exploits the strong linear relationship between relative load (%1RM) and the mean concentric velocity of a given exercise (González-Badillo and Sánchez-Medina, 2010) to enable real-time intensity adjustments (Zhang et al., 2023), effectively resolving the limitations of Percentage-Based Training (PBT). Indeed, recent evidence demonstrates that compared with PBT, VBT can induce greater improvements in lower-limb power and sport-specific performance (Chung et al., 2025). The mechanisms underpinning this advantage have been further elucidated in recent work on elite strength athletes: when total training volume and load are matched, performing repetitions with maximal intended movement velocity (ballistic intent) not only determines the direction of adaptation but also directly drives rapid increases in mean propulsive power (Lecce et al., 2025b), peak propulsive power and rate of force development (RFD) (Lecce et al., 2025c).

Within VBT, the velocity loss threshold (VLT) is a key variable for regulating training intensity and fatigue. During RT, the progressive decline in movement velocity is closely associated with neuromuscular fatigue and reductions in performance (Morán-Navarro et al., 2017). Accordingly, VLT can be used to monitor the degree of fatigue, providing athletes with real-time feedback during training and helping to avoid excessive fatigue and non-productive work. Recent systematic reviews and meta-analyses synthesizing the effects of different VLT have shown that VLT does not appear to substantially influence gains in maximal strength or muscular endurance; however, higher VLT are associated with a greater number of repetitions per set, higher training volume, increased metabolic stress, and higher perceived fatigue, whereas lower VLT, by allowing higher movement velocities and power outputs, seem to be more conducive to improvements in sprint and jump performance (Hernandez-Belmonte and Pallares, 2022; Jukic et al., 2023; Włodarczyk et al., 2021). Similarly, an experimental study in a concurrent training setting (simultaneous strength and endurance training) reported that the use of a higher VLT (45%), although markedly increasing training volume and fatigue, conferred only limited advantages for strength and endurance performance, whereas a lower VLT (15%) elicited favorable adaptations despite a lower training volume (Sánchez-Moreno et al., 2021). Generally, high VLTs favor muscle hypertrophy, whereas lower VLTs optimize explosive performance, a distinction critical for sprinters.

At present, VBT research based on VLT has gradually expanded from adult to youth populations. For example, Rojas-Jaramillo et al. (2024) examined young soccer players and compared the effects of squat training prescribed with VLT of 10% and 30%. Their results showed that, although the 30% VLT group accumulated a higher total training volume, the 10% VLT group achieved greater improvements in 20-m sprint performance and countermovement jump (CMJ) height. While these findings preliminarily support low-VLT strategies for youth, adolescent athletes possess distinct maturing neuromuscular systems and fatigue profiles compared to adults (Granacher et al., 2016). Consequently, identifying the optimal VLT specifically for adolescent sprinters is critical to optimize training efficiency and minimize injury risk through scientific load management.

Specifically, existing youth VBT studies have focused predominantly on team sports rather than sprinting, and none have systematically compared multiple VLTs. Accordingly, this study examined the effects of different VLTs (10%, 20%, and 30%) on lower-limb explosive performance and fatigue in adolescent sprinters during a 6-week intervention.

## 2 Methods

### 2.1 Participants

The sample size was preestimated via G*Power 3.1 software (Dusseldorf, Germany). We set the effect size at f = 0.25, with α = 0.05, and power (1-β) = 0.8. The estimation indicated that a minimum of 42 participants was required for this study. Finally, 45 healthy and active males with at least 3 years of RT experience (Table 1) volunteered to participate and completed the intervention A subsequent *post hoc* power analysis verified that our final sample size of N = 45 provided 83% power to detect a medium effect size (f = 0.25) at α = 0.05. The inclusion criteria for participants were as follows: ① Achieved a sprint performance level equivalent to or above the national second-class athlete standard (Table 2); ② Possessed at least 2 years of resistance training experience, demonstrated proficiency in the free-weight barbell back squat technique, and could perform a back squat with a load equivalent to 1.5 times their body weight; ③ No major injuries within the preceding 6 months; ④ Voluntary participation with full compliance to experimental arrangements. All participants were informed of the study protocol, potential risks, and research objectives prior to participation. Written informed consent was obtained from each participant. The study was conducted in accordance with the Declaration of Helsinki and was approved by the Ethics Committee of Chengdu Sport University (Approval: No. [2025]209).

**TABLE 1:** Basic characteristics of the participants.

Characteristics	VLT10%	VLT20%	VLT30%	P_1-2_	P_1-3_	P_2-3_
Age (years)	16.87 ± 0.83	17.07 ± 0.79	16.93 ± 0.96	0.53	0.83	0.68
Height (cm)	180.47 ± 4.64	179.60 ± 4.95	179.87 ± 4.32	0.61	0.73	0.88
Weight (kg)	67.97 ± 4.97	67.03 ± 5.69	67.23 ± 5.13	0.63	0.70	0.92
Maturity offset	0.69 ± 1.05	0.34 ± 0.82	0.52 ± 0.70	0.82	1.00	1.00
Barbel back squat 1RM (kg)	113.33 ± 11.90	110.80 ± 11.03	111.53 ± 9.79	0.52	0.64	0.86
100 m Personal Best(s)	11.21 ± 0.20	11.28 ± 0.18	11.27 ± 0.17	0.81	0.91	1.00
CMJ-H (cm)	44.14 ± 2.21	43.57 ± 4.05	43.67 ± 3.36	0.64	0.7	0.93
CMJ-RPP (W/kg)	42.71 ± 6.76	41.77 ± 5.69	42.24 ± 3.51	0.64	0.82	0.81
30 m sprint(s)	4.13 ± 0.08	4.16 ± 0.06	4.15 ± 0.03	0.21	0.41	0.65
RSI	2.34 ± 0.45	2.31 ± 0.44	2.32 ± 0.45	0.84	0.88	0.96

P_1–2_ represents the *p*-value between the VLT10% and VLT20% groups; P_1–3_ represents the *p*-value between the VLT10% and VLT30% groups; P_2–3_ represents the *p*-value between the VLT20% and VLT30% groups. A *p* > 0.05 indicates no significant difference between the two groups.

**TABLE 2:** Classification criteria for men’s 100-m sprint performance in China (Electronic Timing).

International elite athlete (s)	National master athlete (s)	National first-class athlete (s)	National second-class athlete (s)	National third-grade athlete (s)
10.25	10.5	10.88	11.54	12.55

Given that the participants in this study were adolescent athletes, To account for the influence of growth and development on performance adaptations, the biological maturation status of the participants was assessed. Biological maturation status was estimated using the Mirwald maturity offset equation, which predicts years from peak height velocity (PHV) based on chronological age, standing height, sitting height, leg length, and body mass (Mirwald et al., 2002). The mean maturity offset (years from PHV) of the participants in this study is presented in Table 1, indicating that most athletes were in a post-PHV stage.

### 2.2 Experimental design

**FIGURE 1:**
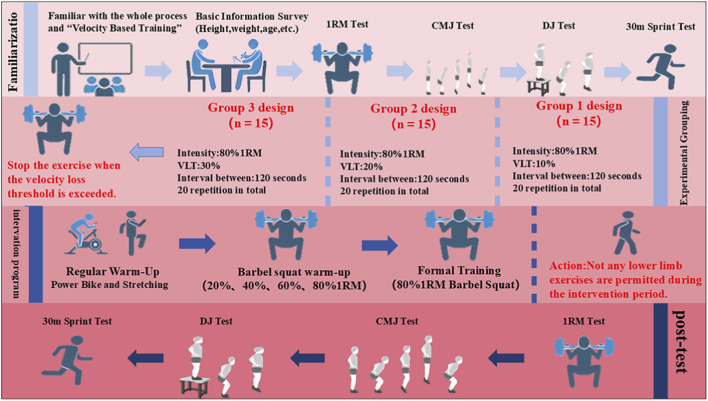
Experimental design overview.

This study adopted a randomized parallel-controlled design to compare the effects of different VLT during VBT on improvements in one-repetition maximum (1RM) barbell back squat, 30 m sprint performance, countermovement jump (CMJ), and drop jump (DJ). A total of 45 adolescent sprinters were randomly assigned in a 1:1:1 ratio to one of three groups using a drawing-of-lots procedure: Experimental Group 1 (VLT10%, n = 15), Experimental Group 2 (VLT20%, n = 15), and Experimental Group 3 (VLT30%, n = 15). Group labels were written on identical slips of paper and placed in an opaque container by an investigator who was not involved in participant recruitment, testing, or data analysis. For each participant, one slip was drawn to determine group allocation, and the allocation was not disclosed to the testing staff until all baseline assessments had been completed. The only difference among the groups was the percentage of allowed VLT during repetitions. All participants performed barbell back squat training twice per week (with a 72-h interval) for six consecutive weeks. The training load, number of repetitions, and inter-set rest intervals were identical across all groups. To minimize the influence of temporal factors, each participant trained on fixed weekdays (Monday/Thursday or Tuesday/Friday) and at consistent times (with a permissible variation of ±1 h). To prevent confounding effects from additional physical exertion, participants were instructed to refrain from engaging in any other high-intensity physical activities, sports training, or competitions during the study period. Baseline testing was conducted 1 week prior to the intervention and required two laboratory visits. During the first visit, participants completed measurements of height, body mass, CMJ, DJ, and 30 m sprint performance. The second visit involved 1RM barbell back squat testing and evaluation of squat velocity at 80% 1RM. All assessments were conducted by the same testers, under identical environmental conditions and at consistent times of day. Before testing, participants performed a standardized warm-up under the supervision of the research staff. A 48-h interval was maintained between the two baseline sessions to avoid fatigue-related effects. Seventy-two hours after the completion of baseline testing, the 6-week intervention commenced. Post-intervention testing was conducted 72 h after the final training session, following the same procedures, order, and 48-h interval as the baseline assessments. The experimental design is shown in Figure 1.

### 2.3 Testing procedures

#### 2.3.1 Counter movement jump (CMJ)

Before the experiment, the researcher calibrated the three-dimensional force platform (KISTLER 9286AA) and established individual participant profiles, recording basic information such as age, sex, height, and body mass. Body mass was recalibrated prior to each testing session. The testing procedure was as follows: upon hearing the commands “step on,” “ready,” “jump,” and “step down,” participants performed the CMJ in sequence. After stepping onto the platform, participants stood with their feet shoulder-width apart and hands placed on the hips. During the “ready” phase, they took a deep breath. At the “jump” command, participants performed a rapid downward movement to a self-selected depth, then immediately executed a maximal vertical jump while maintaining an upright posture, fully extending both legs, and keeping their arms on the hips to avoid rotational movement. Upon landing, they flexed their knees slightly to cushion the impact for 1–2 s (Harman et al., 1990). Each participant performed three CMJ trials with a 60-s rest interval between attempts. Jump height, take-off velocity, and relative peak power were recorded, and the mean of the three trials was used for subsequent data analysis.

#### 2.3.2 Drop jump (DJ)

The force platform operation followed the standardized protocol for the DJ test, which consisted of four verbal commands: “step onto the box,” “ready,” “jump,” and “step off the platform.” Upon hearing the first command, participants stepped onto the box and positioned their feet near the edge, keeping their hands on their hips. At the “ready” command, they raised the non-dominant leg and maintained a single-leg stance. When the “jump” command was given, participants stepped off the box with their dominant leg, landed with both feet on the force platform, and immediately performed a maximal vertical jump upon ground contact. During landing, participants flexed their knees slightly to cushion the impact for 1–2 s. After the “step off the platform” command, both feet left the platform sequentially. Each participant performed three trials with a 60-s rest interval between attempts. The reactive strength index (RSI) was recorded and calculated using the following formula: $RSI=\frac{H}{TC}$ (Young et al., 1995). In this formula, *H* represents the jump height, and *TC* denotes the ground contact time. The mean values obtained from the three trials were used for subsequent data analysis.

#### 2.3.3 30-m sprint test

The 30 m sprint test was conducted using an automatic split-timing system (Smart Speed, Fusion Sport, Australia). During the test, photocell gates were positioned at the starting line and at the 30 m mark, both set at a height of 0.75 m. Participants first performed one familiarization trial, followed by two formal test trials, with a 4-min rest interval between each trial. For data analysis, the mean value of the two sprint times was used as the final result.

#### 2.3.4 Squat 1RM test

Before the start of the experiment, the researchers adjusted the barbell load according to each participant’s actual strength level and ensured that a spotter was positioned behind the participant throughout the test to guarantee safety. The participants were instructed to place the barbell stably across the upper back and shoulders, adjusting it to a comfortable and secure position while elevating the elbows so that the musculature of the upper back and shoulders formed a stable “support shelf” for the barbell. During the descent phase of the squat, participants were required to maintain an upright torso, keep the elbows elevated, and lift and open the chest, while ensuring that the knees tracked in line with the toes. The hips and knees were flexed until the thighs were parallel to the ground. During the ascent phase, participants extended the knees, hips, and torso while maintaining a neutral spine. The 1RM load achieved during the back squat was recorded and used for subsequent data analysis.

#### 2.3.5 Velocity of squat exercise performed at 80% of 1RM

After completion of the 1RM back squat test, participants were given sufficient rest before performing the 80% 1RM squat velocity test. Each participant completed three repetitions, executing the concentric phase of the movement at maximal voluntary speed. The highest velocity achieved across the three trials was recorded. This procedure was used to monitor the participant’s neuromuscular state on the training day and to allow timely adjustment of training loads, thereby facilitating individualized load prescription.

#### 2.3.6 Session-RPE (sRPE) training load

To quantify the internal load of each training session, this study employed the session rating of perceived exertion (sRPE) method based on the Borg CR-10 scale (Foster et al., 2001). Within 30 min after each training session, the same researcher presented participants with the standardized CR-10 scale (Borg, 1982), and instructed them to rate their overall perceived exertion for that session on a scale from 0 to 10, where 0 represented “rest” and 10 represented “maximum effort.” The sRPE load for each session was then calculated by multiplying the reported RPE value by the session’s net duration (in minutes, measured from the end of the specific warm-up to the completion of the final training set), and expressed in arbitrary units (A.U.) (Haddad et al., 2017). Throughout the 6-week intervention period, all participants’ sRPE values were recorded after every training session, and weekly as well as overall mean sRPE values were computed to assess cumulative fatigue across different VLT. This method has been demonstrated to be a valid and reliable tool for monitoring resistance training load (Sweet et al., 2004).

### 2.4 Training program

In this experiment, free-weight barbell back squats were selected as the primary training intervention, performed at an intensity of 80% of 1RM with a total training volume of 20 repetitions per session (Ratamess et al., 2009). The intervention was carried out twice per week. Barbell displacement and velocity were recorded at 50 Hz using a linear position transducer (GymAware RS, Kinetic Performance Technology, Canberra, Australia). The data were processed using the manufacturer’s internal algorithms, and no additional filtering was applied (Oleksy et al., 2023). The system also provided real-time velocity feedback during each training session. Mean velocity (MV) was used as the primary velocity metric to monitor performance and to calculate VLT within each set (Grgic et al., 2020). VLT was defined as the percentage decrement in MV from the fastest (usually the first) to the slowest (last) repetition of each set (Jukic et al., 2023), with the VLT of each repetition expressed as the percentage decrease in MV relative to the MV of the first valid repetition of the set.

Seventy-two hours after completing all pretests, participants began the experimental intervention. Before each training session, participants performed a standardized general warm-up followed by a specific warm-up for the back squat under the supervision of the research staff. During the specific warm-up, the movement velocity at 80%1RM was recorded and compared with the participant’s best velocity obtained during the initial 80%1RM squat velocity test. If the day’s optimal velocity differed by ±0.06 m/s from the reference value, the training load was adjusted by ±5% of 1RM accordingly. This load-adjustment strategy was consistent with the approach adopted in previous studies (Orange et al., 2019).

Before the intervention, the researchers assigned participants to their respective experimental groups and established the corresponding VLT. Participants then performed back squats at maximal intended concentric velocity. The velocity of the first repetition in each set was used as the reference value for monitoring velocity loss and calculating the target velocity range. Each set continued until the squat velocity dropped beyond the preset threshold or fell below the target range. For Experimental Group 1, the VLT was set at 10%. Once a 10% reduction in squat velocity was detected during repetitions, the exercise was immediately terminated, followed by a 2-min rest interval before commencing the next set. This process was repeated until a total of 20 repetitions had been completed, marking the end of the training session. Experimental Groups 2 and 3 followed the same protocol, with VLT set at 20% and 30%, respectively, and the session concluding once the total number of squats reached 20 repetitions. Record the mean number of sets, mean repetitions per set, initial mean velocity, and actual velocity loss (Table 3). If the group’s best movement velocity deviated by ±0.06 m/s from the optimal daily velocity measured at 80% 1RM, the subsequent training load was adjusted by ±5% of 1RM accordingly (Orange et al., 2019). No fixed number of sets or repetitions per set was predetermined; training was terminated upon reaching the designated VLT. The inter-set rest interval was standardized at 2 min, and the post-test assessments were conducted after the completion of all 20 repetitions.

**TABLE 3:** Training characteristics of 80% 1RM back squats in different groups.

Group	Mean number of sets	Mean repetitions per set	Initial mean velocity	Actual velocity loss
VLT 10%	5.8 ± 1.0	3.6 ± 0.8	0.72 ± 0.08	12.8 ± 3.4
VLT 20%	4.4 ± 0.8	4.6 ± 1.1	0.69 ± 0.06	21.5 ± 2.6
VLT 30%	3.0 ± 0.6	6.8 ± 1.4	0.70 ± 0.07	32.2 ± 3.7

### 2.5 Statistical analyses

All test data were entered and organized using Excel, and statistical analyses were performed with SPSS 27.0 (IBM Corp., Armonk, NY, United States). Homogeneity of variance was evaluated using Levene’s test (p ≥ 0.05), and normality was examined using the Shapiro–Wilk test (p ≥ 0.05); all variables satisfied the assumptions of homogeneity of variance and normal distribution.

Baseline characteristics and pre-test values were first compared using a one-way analysis of variance (ANOVA) to assess equivalence among the three groups. To robustly assess the effects of the intervention, a one-way analysis of covariance (ANCOVA) was employed for all performance variables to analyze between-group differences at post-test. In this model, pre-test values and biological maturity were entered as covariates. This approach was chosen to strictly account for baseline differences and the potential confounding influence of maturation. Additionally, paired-samples t-tests were conducted to verify within-group changes from pre-to post-test. For fatigue levels (measured during the intervention), a one-way ANOVA was used to compare differences among the experimental groups. For the ANCOVA and one-way ANOVA, when a significant main effect was detected, Bonferroni-adjusted *post hoc* tests were applied to identify specific between-group differences. Effect sizes (ES) were calculated to determine the practical magnitude of the findings. Partial eta squared (partial η^2^) was used to evaluate the overall main effect of Group in the ANCOVA and ANOVA models, with values classified as small (0.01 < partial η^2^ ≤ 0.06), medium (0.06 < partial η^2^ ≤ 0.14) and large (partial η^2^ > 0.14). For all pairwise comparisons, including within-group changes (Pre vs. Post) and specific between-group differences (e.g.,,VLT10 vs. VLT30), Hedges’ g was calculated. Values for Hedges’ g > 0.50 were considered moderate and > 0.80 were considered large.

To explicitly evaluate the practical relevance of the findings, 95% confidence intervals (95% CIs) were calculated for all pairwise mean differences and Hedges’ g effect sizes. The level of statistical significance was set at p < 0.05, and exact p-values are reported for all outcomes. Reliability was assessed using intraclass correlation coefficients (ICC), with ICC ≥ 0.75 indicating high reliability.

## 3 Results

### 3.1 Reliability of measurements

**FIGURE 2:**
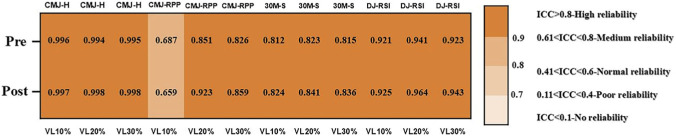
Test Reliability. Note: CMJ-H = countermovement jump height; CMJ-RPP = countermovement jump relative peak power; 30M-S = 30 m sprint time; DJ-RSI = drop jump reactive strength index.

To ensure the reliability of the tests, the research team calculated the intraclass correlation coefficients (ICCs) for each group at different time points. The results showed that the ICCs for CMJ height, 30 m sprint, and DJ reactive strength index (RSI) in all groups were above 0.75, meeting the criterion for high reliability. Additionally, the ICCs for CMJ relative peak power in all groups exceeded 0.60, indicating moderate reliability (Figure 2).

### 3.2 Maximum strength

Following the 6-week experimental intervention, highly significant improvements from pre-to post-test were observed across all measured velocities (VLT10%: 113.33 ± 11.90 vs. 120.80 ± 7.74, p = 0.006, Hedge’s g = 1.24; VLT20%: 110.67 ± 11.16 vs. 115.87 ± 8.32, p = 0.009, Hedge’s g = 1.13) and significant improvements was observed in VLT30% (111.34 ± 9.90 vs. 116.07 ± 6.79, p = 0.013, Hedge’s g = 0.90) (Table 4).

**TABLE 4:** Descriptive statistics and within-group changes from pre-test to post-test across different VLT.

Variables	Group	Pre	Post	Mean difference (95% CI)	P	Hedge’s g (95%CI)
Back squat 1RM (kg)	10%	113.33 ± 11.90	120.80 ± 7.74	−7.47 (−10.72, −4.22)	0.006	−1.24 (−1.90, −0.56)
	20%	110.67 ± 11.16	115.87 ± 8.32	−5.20 (−7.68, −2.72)	0.009	−1.13 (−1.76, −0.48)
	30%	111.34 ± 9.90	116.07 ± 6.79	−4.73 (−7.58, −1.88)	0.013	−0.90 (−1.48, −0.30)
CMJ-H (cm)	10%	44.09 ± 2.20	49.02 ± 4.87	−4.91 (−7.17, −2.65)	0.001	−1.17 (−1.81, −0.51)
	20%	43.57 ± 4.05	47.01 ± 3.31	−3.43 (−5.14, −1.72)	0.003	−1.08 (−1.70, −0.44)
	30%	43.67 ± 3.36	45.19 ± 3.46	−1.51 (−2.81, −0.21)	0.014	−0.63 (−1.16, −0.08)
CMJ-RPP (W/kg)	10%	42.71 ± 6.76	47.77 ± 7.45	−5.06 (−7.43, −2.68)	0.002	−1.15 (−1.78, −0.49)
	20%	41.77 ± 5.69	45.64 ± 5.30	−3.87 (−6.08, −1.66)	0.008	−0.94 (−1.53, −0.33)
	30%	42.24 ± 3.51	44.33 ± 4.68	−2.09 (−3.55, −0.62)	0.015	−0.77 (−1.32, −0.19)
30 m sprint(s)	10%	4.13 ± 0.08	4.07 ± 0.06	0.07 (0.04, 0.10)	0.001	1.25 (0.56, 1.91)
	20%	4.16 ± 0.06	4.12 ± 0.07	0.04 (0.02, 0.06)	0.005	1.15 (0.49, 1.80)
	30%	4.15 ± 0.03	4.13 ± 0.03	0.02 (0.01, 0.03)	0.015	0.93 (0.32, 1.52)
RSI (m/s)	10%	2.34 ± 0.45	2.55 ± 0.35	−0.20 (−0.31, −0.10)	0.016	−1.04 (−1.65, −0.40)
	20%	2.31 ± 0.44	2.43 ± 0.36	−0.11 (−0.18, −0.05)	0.018	−0.90 (−1.49, −0.30)
	30%	2.32 ± 0.45	2.39 ± 0.38	−0.07 (−0.11, −0.02)	0.019	−0.74 (−1.30, −0.17)

Data are presented as Mean ± SD, for “Pre” and “Post” columns. P indicates the p-value for the within-group difference (pre-vs. post-test) based on paired samples T-tests. Hedge’s g (95% CI) represents the within-group effect size.

Regarding Back Squat 1RM, ANCOVA showed no statistically significant differences in post-test scores between groups after controlling for maturity and pre-test scores (F = 3.16, p = 0.53, partial-η^2^ = 0.13). The pre-test score was a highly significant covariate (F = 198.84, p < 0.001, partial-η^2^ = 0.83), whereas maturity was not (F = 2.56, p = 0.12, partial-η^2^ = 0.06) (Table 5).

**TABLE 5:** Results of the one-way analysis of covariance (ANCOVA) examining the effect of VLT on post-test performance, controlling for maturity and pre-test scores.

Variables	F_1_	F_2_	F_3_	P_1_	P_2_	P_3_	ES_1_	ES_2_	ES_3_
Back squat 1RM (kg)	2.56	198.84	3.16	0.12	<0.001	0.53	0.06	0.83	0.13
CMJ-H (cm)	1.83	21.24	5.34	0.18	<0.001	0.01	0.04	0.35	0.21
CMJ-RPP (W/kg)	2.05	65.54	3.20	0.16	<0.001	0.04	0.05	0.62	0.14
30 m sprint(s)	0.34	61.20	8.24	0.57	<0.001	0.01	0.08	0.61	0.30
RSI (m/s)	0.01	375.91	4.26	0.92	<0.001	0.02	0.01	0.90	0.18

F_1_represents the effect of the covariate “Maturity”; F_2_represents the effect of the covariate “Pretest Scores”; F_3_represents the effect of the factor “Group”; P_1_represents the significance test result for the “Maturity” covariate effect; P_2_represents the significance test result for the “Pretest Scores” covariate effect; P_3_represents the significance test result for the “Group” main effect; ES_1_represents the effect size (partial η^2^) of the “Maturity” covariate; ES_2_represents the effect size (partial η^2^) of the “Pretest Scores” covariate; ES_3_represents the effect size (partial η^2^) of the “Group” main effect.

### 3.3 Counter movement jump height

After the 6-week intervention, highly significant improvements from baseline were observed in VLT10% (44.09 ± 2.20 vs. 49.02 ± 4.87, p = 0.001, Hedges’ g = 1.17) and VLT20% (43.57 ± 4.05 vs. 47.01 ± 3.31, p = 0.003, Hedges’ g = 1.08), while VLT30% showed a significant improvement (43.67 ± 3.36 vs. 45.19 ± 3.46, p = 0.014, Hedges’ g = 0.63) (Table 4).

For CMJ jump height, ANCOVA revealed a significant difference in post-test scores between groups after adjusting for maturity and pre-test scores (F = 5.34, p = 0.01, partial-η^2^ = 0.21). Post hoc comparisons indicated that the VLT10% group achieved significantly higher scores than the VLT30% group (p = 0.01). The pre-test score had a highly significant covariate effect (F = 21.24, p < 0.001, partial-η2 = 0.35), whereas the effect of maturity was not significant (F = 1.83, p = 0.18, partial-η2 = 0.04) (Table 5).

### 3.4 Counter movement jump relative peak power

Following the 6 weeks intervention, VLT10% and VLT20% showed a highly significant improvement from pre-test to post-test (VLT10%: 42.71 ± 6.76 vs. 47.77 ± 7.45, p = 0.002, Hedges’ g = 1.15; VLT20%: 41.77 ± 5.69 vs. 45.64 ± 5.30, p = 0.008, Hedges’ g = 0.94) and VLT30% showed a significant improvement (42.24 ± 3.51 vs. 44.33 ± 4.68, p = 0.015, Hedges’ g = 0.77) (Table 4).

For CMJ relative peak power, ANCOVA indicated a significant between-group difference in post-test scores after adjusting for maturity and pre-test scores (F = 3.20, p = 0.04, partial-η^2^ = 0.14). Post hoc comparisons indicated that the post-test score of the VLT10% group was significantly higher than that of the VLT30% group (p = 0.04). The covariate effect of the pre-test score was highly significant (F = 65.54, p < 0.001, partial-η^2^ = 0.62), while the effect of maturity was not significant (F = 2.05, p = 0.16, ES = 0.05) (Table 5).

### 3.5 30-m sprint

The 6-week intervention resulted in significant reductions in 30-m sprint times (indicating improvement) across all groups (Table 6). Specifically, the VLT10% group showed the most substantial decrease (4.13 ± 0.08 vs. 4.07 ± 0.06 s, p = 0.001, Hedges’ g = 1.25), followed by VLT20% (4.16 ± 0.06 vs. 4.12 ± 0.07 s, p = 0.005, Hedges’ g = 1.15) and VLT30% (4.15 ± 0.03 vs. 4.13 ± 0.03 s, p = 0.015, Hedges’ g = 0.93) (Table 4).

**TABLE 6:** Specific warm-up protocol for the squat exercise plan.

Load intensity	Repetitions
20% 1RM	3
40% 1RM	3
60% 1RM	3
80% 1RM	2

For 30-m sprint time, ANCOVA results showed a significant difference in post-test scores between groups after controlling for maturity and pre-test scores (F = 8.24, p = 0.01, partial-η^2^ = 0.30). Post hoc comparisons indicated that the VLT10% group was significantly faster than the VLT30% group (p = 0.01). The pre-test score exerted a significant covariate effect (F = 61.20, p < 0.001, partial-η^2^ = 0.61), whereas the covariate effect of maturity was not significant (F = 0.34, p = 0.57, ES = 0.08) (Table 5).

### 3.6 Drop jump

Following the 6-week intervention, all groups demonstrated significant improvements in reactive strength index from baseline (Table 6). The VLT10% group showed the largest increase (2.34 ± 0.45 to 2.55 ± 0.35, p = 0.016, Hedges’ g = 1.04), with significant gains also observed in the VLT20% (2.31 ± 0.44 vs. 2.43 ± 0.36, p = 0.018, Hedges’ g = 0.90) and VLT30% groups (2.32 ± 0.45 vs. 2.39 ± 0.38, p = 0.019, Hedges’ g = 0.74) (Table 4).

ANCOVA results revealed a significant difference in post-test scores between groups after adjusting for covariates (F = 4.26, p = 0.02, partial-η^2^ = 0.18). Post hoc analysis indicated that the VLT10% group attained significantly higher scores than the VLT30% group (p = 0.02). While the pre-test score was a significant covariate (F = 375.91, p < 0.001, partial-η^2^ = 0.90), maturity was not (F = 0.01, p = 0.92, partial-η^2^ = 0.01) (Table 5).

### 3.7 Session-RPE (sRPE) training load

**FIGURE 3:**
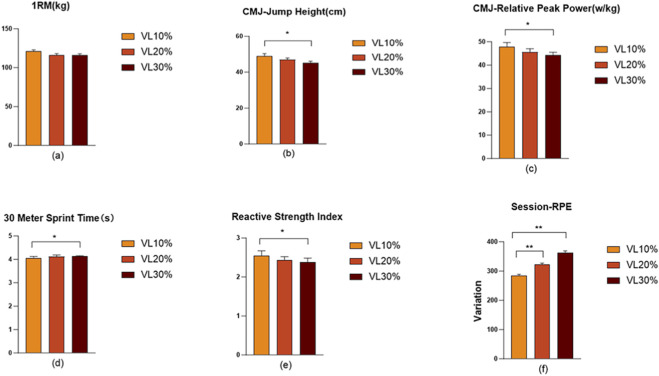
Improvements in Test Parameters Across Experimental Groups. Note: **(a–e)** Represent the post-intervention test values of the three groups after 6 weeks; **(a)** Post-test 1RM squat value; **(b)** Post-test countermovement jump (CMJ) height; **(c)** Post-test CMJ relative peak power; **(d)** Post-test 30-m sprint performance; **(e)** Post-test reactive strength index (RSI); **(f)** sRPE comparison among the three groups.

To evaluate subjective fatigue, sRPE training load was monitored throughout the 6-week intervention. One-way ANOVA revealed a significant difference in accumulated sRPE values among the three groups (*p* < 0.001). Post-hoc analysis showed that the VLT%10 had significantly lower sRPE values (284.39 ± 18.29 A.U.) compared with both the VLT20% group (322.17 ± 19.03 A.U.) and the VLT30% (363.64 ± 21.86 A.U.) (*p* < 0.001). Additionally, the VLT20% exhibited significantly lower sRPE values than the VLT30% (*p* < 0.001). These findings indicate that, under the same total training volume, a higher VLT leads to greater accumulated subjective fatigue, demonstrating a clear dose–response relationship (Figure 3).

## 4 Discussion

The primary purpose of this study was to examine the effects of VBT with different VLT on lower-limb explosive performance in adolescent sprinters. The results demonstrated that all VBT protocols effectively improved athletic performance; however, the lower VLT (10%) induced superior adaptations in most explosive performance indicators, while also resulting in lower accumulated fatigue.

### 4.1 Effects of VBT on squat 1RM performance

After 6 weeks of experimental intervention, significant improvements in 1RM squat performance were observed across all experimental groups, with no statistical differences in the magnitude of improvements between groups. Furthermore, the order of improvement was 10%VLT > 20%VLT > 30%VLT. This suggests that, under a fixed training volume (20 repetitions), the VBT protocols at three different VLT yield similar effects on maximal strength. This phenomenon can be attributed to several factors: Firstly, this study employed high load intensity (80% 1RM), which has been shown to promote greater neural adaptations compared to moderate or low loads, thereby optimizing strength gains (Androulakis-Korakakis et al., 2020; Moss et al., 1997); Secondly, VBT requires participants to complete each concentric phase as quickly as possible, emphasizing the high activation of the nervous system in relation to movement speed. Motor units, consisting of a single alpha motor neuron and all the muscle fibers it innervates, produce force and contraction speed based on the number and type of motor units recruited. Henneman et al. (1965) demonstrated that motor unit recruitment follows a “size principle,” meaning that higher force and power demands gradually recruit larger motor units, predominantly composed of fast-twitch IIa and IIx fibers. Therefore, in training aimed at enhancing explosiveness, deliberately prioritizing fast force production and the recruitment of high-threshold motor units is of significant importance (Pareja-Blanco et al., 2014). From a neuromuscular perspective, VBT combines high loads with fast velocities, providing a strong stimulus to the nervous system. This stimulus promotes the optimal recruitment and synchronization of motor units (prioritizing high-threshold II-type fast-twitch fibers) and may alleviate neuromuscular inhibition through high-intensity training (Aagaard et al., 2000; 2002).

The participants in the present study were adolescent athletes, whose physiological adaptation mechanisms differ substantially from those of adults. This may be a key reason why the low VLT (10%) condition exhibited superior outcomes. Adolescents are in a critical period of neural development and myelination and thus possess heightened neural plasticity (Myer et al., 2013). Compared with adults, strength gains in youth are more strongly driven by neural adaptations, particularly motor unit recruitment, activation, and synchronization (Behm et al., 2008), which makes their responsiveness to training that targets neuromuscular plasticity more pronounced. Consequently, they tend to benefit markedly from training protocols characterized by high intensity and high power output that are specifically designed to optimize neural system development. In addition, for adolescents whose nervous systems are not yet fully mature, fatigue management is of paramount importance. The high level of fatigue induced by 30% VLT may disrupt correct movement patterns and thereby attenuate optimal neural adaptations (Branscheidt et al., 2019). In contrast, the low-fatigue environment associated with 10% VLT allows each repetition to be performed with high quality and high neural drive. Such high-quality, high-drive repetitions, through repeated practice, help to promote central neural adaptations and constitute a key condition for enhancing the adaptive capacity of the nervous system (Gabriel et al., 2006). High-quality repetitive practice performed under low-fatigue conditions is considered a critical factor for inducing rapid functional plasticity in the motor cortex (Classen et al., 1998). Given that the cerebral cortex of adolescents is at a peak of plasticity, this training strategy may amplify the neural adaptation benefits of strength training to an even greater extent than in adults.

### 4.2 Effects of VBT on 30-m sprint performance

After the 6-week VBT intervention, 30-m sprint performance improved significantly in all experimental groups, and the magnitude of improvement followed a clear pattern of 10% VLT > 20% VLT > 30% VLT, with the 10% VLT group performing significantly better than the 30% VLT group. These findings demonstrate that VBT can effectively enhance sprint speed in sprinters, and that lower VLTs are more advantageous. The improvement in sprint ability can be primarily attributed to increases in lower-limb maximal strength. Athletes with greater lower-limb strength are able to generate higher ground reaction forces within very short ground contact times, thereby increasing step frequency and step length, and ultimately improving sprint speed (Cometti et al., 2001; Seitz et al., 2014; Weyand et al., 2000). During adolescence, surges in androgens and growth hormone make this a critical period for the development of type II muscle fibers, muscle cross-sectional area, and neuromuscular coordination (Beunen and Thomis, 2000; Evans and Lexell, 1995), providing an optimal window for the development of speed qualities. More importantly, the rate of force development (RFD) reflects the capacity to generate force rapidly and is a key determinant of initial acceleration performance (Folland et al., 2014; Weyand et al., 2010). Maximal strength is closely associated with RFD, and increases in lower-limb strength are often accompanied by improvements in RFD (Maffiuletti et al., 2016).

In the present study, lower VLT (e.g., 10%) effectively limited the number of repetitions performed within each set, thereby controlling the accumulation of neuromuscular fatigue. As a result, athletes were able to maintain very high concentric velocities and power outputs for the majority of training repetitions (Pérez-Castilla and García-Ramos, 2020; Weakley et al., 2020). At the same time, a low-fatigue environment is conducive to increasing motor unit firing rates (Van Cutsem et al., 1998), thereby taking advantage of the excellent neural plasticity observed in adolescents to more effectively establish and consolidate neuromuscular patterns associated with rapid force production (Laube et al., 2020). For sprint performance, this high-frequency, rapidly recruited force-production pattern is highly specific to the demands of the sport. Conversely, higher VLTs (e.g., 30%) are more likely to induce substantial fatigue and lead to marked decrements in neural function. For adolescents whose motor control systems are still undergoing refinement, such fatigue may disrupt the formation of sprint-specific rapid force-production patterns, interfere with the development of speed-specific neural pathways, and ultimately limit the efficiency with which training adaptations transfer to sprint performance (Rodríguez-Rosell et al., 2021).

It is noteworthy that not all studies have supported the efficacy of VBT for improving sprint performance. Orange et al. (Orange et al., 2019) compared the effects of a 7-week in-season VBT program with PBT on strength, jumping, and sprint performance in elite youth rugby players competing in the English Premiership. Their results showed that both training methods significantly increased 1RM strength and lower-limb power; however, sprint performance declined in both groups. The authors suggested that this phenomenon might be attributable to two main factors. First, the training program did not include any linear sprint drills or horizontally oriented resisted exercises (e.g., acceleration sprints, sled towing), thereby failing to provide velocity-specific neuromuscular stimuli. Second, all participants were professional athletes tested during the competitive season; in addition to resistance training, they were exposed to high loads of match play and skill training, and the cumulative fatigue may have masked potential speed adaptations. In contrast to that study, the VBT intervention in the present work was conducted during a non-competitive period, when participants were not subjected to heavy match demands, and their sport-specific training incorporated a large volume of sprint work. This context likely allowed for more robust neuromuscular adaptations in speed, strength, and coordination. The VBT model employed here, characterized by high movement velocities and low accumulated fatigue, combined with frequent, horizontally oriented sprint training in the athletes’ sport-specific sessions, may represent a key factor underpinning the substantial improvements in sprint performance observed in our participants. These findings suggest that the ability of VBT to enhance sprint performance is highly dependent on the training model and implementation context. When VBT is applied in-season under conditions of high accumulated fatigue and with limited exposure to sprint-specific training, sprint performance may fail to improve or may even decline. Conversely, in a training environment with well-managed fatigue and strong velocity specificity, particularly when low velocity loss thresholds are used, VBT appears to be effective for substantially improving short-distance sprint performance. This highlights the need for future training designs to integrate considerations of training phase, movement direction, and fatigue management in order to leverage VBT optimally and maximize improvements in speed performance.

### 4.3 Effects of VBT on counter movement jump performance

After the 6-week intervention, all experimental groups showed significant improvements in jump height and relative peak power in the CMJ test, with the magnitude of improvement following the pattern 10% VLT > 20% VLT > 30% VLT, and the 10% VLT group exhibiting significantly greater gains than the 30% VLT group. These findings confirm that VBT is an effective method for developing vertical explosive performance, with a clear advantage for lower VLT. The enhancement in CMJ performance can be attributed first to increases in lower-limb strength. Athletes with greater lower-limb strength are able to produce a larger impulse under the force–time curve during take-off, thereby achieving greater jump height and higher power output (Cormie et al., 2010). In addition, the training exercise (free-weight back squat) and the testing task (CMJ) are highly similar in their biomechanical structure: both involve utilization of the stretch–shortening cycle (SSC) and are characterized by a predominantly vertical force-production pattern. This high degree of specificity likely facilitated the positive transfer of training adaptations to CMJ performance (Sleivert and Taingahue, 2004).

The effectiveness of VBT lies in its emphasis on movement velocity. Maximizing execution speed during resistance training can optimize motor unit recruitment and firing frequency (Cormie et al., 2010), thereby ensuring that the nervous system drives the musculature efficiently. This mechanism is particularly critical in adolescents, as gains in explosive strength in this population are predominantly mediated by neural adaptations (Faigenbaum et al., 2009). More specifically, the neuromuscular adaptations induced by VBT are closely linked to the selected VLT. Under a low VLT (10%), fatigue is strictly controlled, allowing athletes to perform repetitions at or near optimal velocity more frequently. From the perspective of adolescent neural development, such high-quality repetitions maximize the effectiveness of the stretch–shortening cycle (SSC). Each high-velocity, low-fatigue contraction reinforces the sensitivity of the stretch reflex and the efficiency of muscle spindle feedback (Lloyd et al., 2014). This process enhances the ability of the neuromuscular system to generate powerful concentric contractions immediately following eccentric loading and markedly increases motor unit firing rates. In contrast, the greater fatigue accumulated under a 30% VLT not only reduces movement velocity during training but may also hinder the optimization of these reflex mechanisms. Given that the nervous system of adolescent athletes is still maturing, such fatigue may augment protective neural inhibition, increasing the sensitivity of the Golgi tendon organs (Avela et al., 1999), thereby attenuating the recruitment of high-threshold motor units. This may help explain why the CMJ improvements in the high-VLT condition were comparatively smaller.

### 4.4 Effects of VBT on drop jump performance

After 6 weeks of experimental intervention, significant improvements in drop jump performance, as indicated by the Reactive Strength Index (RSI), were observed in all groups, showing a trend of 10% VL > 20% VL > 30% VL. This suggests that Velocity-Based Training (VBT) effectively enhances athletes’ performance in supramaximal eccentric exercises, with the low VL threshold strategy demonstrating a particularly pronounced advantage.

The optimization of the RSI heavily relies on the nervous system’s ability to organize efficient force production patterns within extremely short timeframes. Adolescence is considered a critical window for tendon stiffness adaptation (Mersmann et al., 2017), and low VL threshold training creates an ideal physiological environment for inducing such adaptations by maintaining high movement velocities and minimizing fatigue. In this environment, the synchronization of motor units and the disinhibition of neuromuscular control are maximized (Milner-Brown and Stein, 1975; Semmler and Nordstrom, 1998). This is particularly crucial for adolescents, whose inhibitory systems are still developing and are more susceptible to fatigue-induced protective inhibition (Pascual-Leone et al., 2011). The low-fatigue stimulus of the 10% VL group allows the nervous system to gradually adapt to high-intensity rapid loading, optimizing the utilization efficiency of tendon elasticity, enhancing lower limb stiffness, and enabling athletes to generate smaller joint deformations upon landing and take off more quickly. In contrast, the accumulated fatigue from higher VL thresholds compromises this fine-tuned neural control, leading to prolonged ground contact times and limiting RSI improvements.

The improvement in explosive strength largely depends on the enhancement of Common Synaptic Input (CSI). Research by Lecce , et al. (2025a) suggests that the increase in strength performance is not primarily driven by changes at the individual muscle level, but rather by an increase in the “Net Excitatory Drive” from the brain to the spinal motor neurons. The enhanced common input leads to a series of key neuroadaptive changes: ① decreased motor unit recruitment thresholds; ② promotion of more coordinated and regular discharge patterns; ③ increased CSI proportion; and ④ reduced fluctuations in common input. In this study, the advantage of the 10% VLT group lies in its ability to effectively avoid the accumulation of severe metabolic fatigue, which can diminish the strength and clarity of common synaptic input by increasing the “noise” in afferent neural feedback, leading to desynchronization of motor unit discharges. For adolescent athletes, the 10% VLT training, with its high-frequency, brief, and minimally disruptive high-quality stimuli, enhances the transmission efficiency of the corticospinal pathway, leading to more consistent discharge activity in spinal anterior horn motor neurons during explosive force tasks, thereby directly improving RSI performance.

It is important to note that the improvement in RSI is not an isolated phenomenon, but is highly consistent with the overall improvement in sprinting and vertical jump performance observed in this study. This finding supports the principle of “velocity specificity,” but the underlying mechanism lies not only in fatigue management but also in the strict maintenance of Maximal Intended Velocity (MIV). Recent research has indicated that MIV is a key driver of explosive strength adaptations; training at the maximal intended velocity is the most effective way to promote increases in power and RFD (Lecce et al., 2025b; Lecce et al., 2025c). In the 30% VLT group, the involuntary deceleration in the later stages of the set essentially rewrites a “low-speed program” in the neural pathways, effectively repeating a “suboptimal movement pattern.” In contrast, the 10% VLT protocol ensures that each repetition remains within the effective MIV window by terminating the set immediately as speed begins to decline, thus avoiding inefficient repetitions (Junk Reps) that would disrupt the explosive neuromuscular pattern. This mechanism not only explains the improvement in RSI but also provides a unified physiological explanation for the comprehensive advantage of the low VLT across all explosive performance indicators observed in this study.

It should be noted that Rissanen et al. (2022) reported that higher velocity loss thresholds were associated with superior outcomes in trained female athletes. This discrepancy suggests that individual factors such as sex, training status, and baseline strength may influence the optimal prescription of VLT. In the present study, participants were male adolescent sprinters with an established strength base (back squat 1RM ≈ 1.5 times body mass), whose training adaptation profile may differ substantially from that of the female athletes examined in the aforementioned study. Therefore, it can be inferred that the optimal VLT exhibits a certain degree of population specificity and should be individualized according to the characteristics of different athlete populations.

### 4.5 Limitations

Although this study provides valuable insights into the application of different VLT in VBT for adolescent sprinters, several limitations should be acknowledged when interpreting the findings. First, the study did not include a PBT control group. Therefore, the current results can only confirm that VBT protocols employing different VLT effectively improve athletic performance, but they cannot directly and conclusively demonstrate the superiority of VBT over commonly used PBT methods in youth training. Future research incorporating a PBT control group would enable a more definitive evaluation of VBT’s unique advantages in training precision and efficiency. Second, fatigue monitoring in this study relied primarily on subjective sRPE, without the inclusion of more objective physiological or biochemical markers (e.g., blood or hormonal indices, creatine kinase activity, or heart rate variability), nor direct assessments of neuromuscular fatigue. Third, biological maturity in this study was estimated using maturity offset, a predictive model based on regression equations. Although widely used, this is still an indirect surrogate measure that cannot precisely reflect an individual’s neuroendocrine or skeletal maturity. This may introduce potential bias when controlling for maturity-related influences on training responses. Finally, it is important to note that some of the outcome measures, particularly improvements in jump performance, showed relatively high inter-individual variability (i.e., large standard deviations relative to the mean). Such high variability is typical in adolescent populations and is primarily attributed to inherent differences in individuals’ biological maturation rates, genetic background, and neuromuscular adaptation potential, all of which contribute to significant heterogeneity in training responses. In summary, future studies could incorporate PBT interventions while integrating multidimensional physiological monitoring and more direct measures of biological maturity to further validate and elucidate the mechanisms underlying the effects of different VLT in adolescent athletic populations.

## 5 Conclusion

Over the 6-week intervention period, all three VBT protocols employing different velocity loss thresholds (VLTs of 10%, 20%, and 30%) elicited significant improvements in maximal strength, 30-m sprint performance, CMJ height, CMJ relative peak power, and RSI in male adolescent sprinters. These findings indicate that VBT is an effective strategy for enhancing lower-limb explosive performance in this population. However, the magnitude of adaptation was not uniform across protocols: the 10% VLT group consistently exhibited the greatest improvements in CMJ height, CMJ relative peak power, 30-m sprint performance, and RSI, with gains that were significantly superior to those observed in the 30% VLT group. This pattern suggests that lower VLTs are more favorable for inducing high-quality neuromuscular adaptations while constraining excessive fatigue. In contrast, when total training volume was equated, higher VLT settings were associated with markedly greater sRPE, indicating increased internal load, which may have compromised the quality of individual training sessions and interfered with subsequent recovery.

Mechanistically, the superiority of the low velocity loss protocol (10% VLT) appears to derive from the strict maintenance of MIV and the minimization of neuromuscular fatigue. For adolescent athletes who are in a critical window of neural plasticity, this approach may optimize motor unit synchronization and reinforce high-velocity movement patterns without being compromised by fatigue-induced signal attenuation.

Taken together, the findings of the present study indicate that VBT is an effective strategy for enhancing lower-limb strength and explosive performance in adolescent sprinters, and that the use of relatively low VLT (e.g., 10%) may provide coaches with valuable guidance for maximizing gains in explosive performance while maintaining manageable levels of fatigue. However, these results should be generalized with caution. As biological maturation was estimated via maturity offset rather than directly assessed, the interaction between VBT training load and specific stages of biological maturation remains to be elucidated. Future practical applications should therefore consider athletes’ maturation status in conjunction with velocity-derived metrics. Moreover, the absence of a PBT control group represents a limitation of the present study. Future research should incorporate a PBT comparison group, longer intervention periods, and more comprehensive physiological and biochemical monitoring to verify the current findings and further clarify the mechanisms linking VLT prescription, fatigue management, and training efficiency in adolescent athletes.

## Data Availability

The raw data supporting the conclusions of this article will be made available by the authors, without undue reservation.
